# Intractable middle ear effusion in EGPA patients might cause permanent hearing loss: a case–control study

**DOI:** 10.1186/s13223-022-00706-x

**Published:** 2022-08-06

**Authors:** Noeul Kang, Joongbo Shin, Yang-Sun Cho, Jin-Young Lee, Byung-Jae Lee, Dong-Chull Choi

**Affiliations:** 1 Division of Allergy, Department of Medicine, Samsung Medical Center, Sungkyunkwan University School of Medicine, 81 Irwon-ro, Gangnam-gu, Seoul, 06351 Republic of Korea; 2 Department of Otorhinolaryngology-Head and Neck Surgery, Samsung Medical Center, Sungkyunkwan University School of Medicine, Seoul, Korea; 3 Health Promotion Center, Samsung Medical Center, Seoul, Korea

**Keywords:** Eosinophilic granulomatosis with polyangiitis, Eosinophilic otitis media, Middle ear effusion, Otologic, EGPA, EOM, MEE

## Abstract

**Background:**

Ear, nose, and throat involvement are common in eosinophilic granulomatosis with polyangiitis (EGPA). Among otologic manifestation, middle ear effusion (MEE) is less recognized but a problematic condition as it may progress to hearing impairment when left untreated. This study aimed to evaluate the characteristics, risk factors and clinical outcomes of MEE in EGPA patients.

**Methods:**

This is a case–control study of patients who were diagnosed and treated for EGPA from January 1995 to November 2018. Patients with ear symptoms (ear fullness, ear discharge, tinnitus or hearing loss) were assessed by otologists and were included in the case group (n = 23) if clinically relevant. The other patients without MEE were included in the control group (n = 52). Risk of MEE was calculated using the Cox proportional-hazard model.

**Results:**

During median follow-up of 9.9 years, 23 (30.7%) out of 75 patients had MEE. In MEE group, 12 (52.2%) patients had hearing loss; conductive type in 10 (10/12, 83.3%) and mixed type in two (2/12, 16.7%). In multivariable regression analysis, major organ involvement at diagnosis (adjusted hazard ratio [aHR] 65.4; 95% confidence interval [CI], 1.50—2838.39; *P* = 0.030] , early onset of ear symptom after systemic therapy (< 6 months) (aHR 40.0; 95% CI, 1.35—1183.43; *P* = 0.033) and continuing the maintenance steroid without cessation (aHR 8.59; 95% CI, 1.13—65.42; *P* = 0.038) were independently associated with a risk of MEE. To control MEE, 16 (69.6%) patients had to increase maintenance steroid dose and 9 (39.1%) patients experienced recurrent MEE whenever maintenance dose was tapered.

**Conclusions:**

MEE is a common but frequently neglected condition in EGPA which is often intractable. The maintenance steroid dose should be adequately adjusted to control MEE and to prevent from progressive hearing loss. Novel biologic agents possibly have a role in controlling MEE in EGPA.

## Background

Eosinophilic granulomatosis with polyangiitis (EGPA), previously known as Churg–Strauss syndrome, is a rare systemic disease characterized by eosinophil-rich granulomatous inflammation often involving the respiratory tract [1]. The most commonly used criteria for diagnosis of EGPA is the one suggested by American College of Rheumatology (ACR) in 1990, which requires at least four of the followings: asthma, peripheral blood eosinophilia > 10% on differential count, mono- or poly-neuropathy, transient pulmonary infiltrates, paranasal sinus (PNS) abnormalities, and biopsy of a blood vessel with an accumulation of eosinophils in extravascular area [2].

The ear, nose, and throat involvements are frequent in EGPA. Rhinitis has been reported in 15–75% of total cases, occurring before, or at the onset of asthma. Chronic sinusitis has been reported in 60–80% of cases, and PNS abnormality is accompanied in 75% [1, 3–10]. As for otologic manifestations, hearing loss and middle ear effusion (MEE) are the most common presentation [11]. MEE in EGPA is characterized with thick, mucoid aural discharge with increased number of eosinophils, and conductive hearing loss may result from effusion or obstruction resulting from eosinophilic granuloma [9, 12, 13]. Early recognition of MEE is challenging because ear involvement may occur without concomitant systemic disease activity or life threatening complications [14].

The otologic manifestations of EGPA were reported as rare events, based on a small number of individuals [9]. However, it has recently gained attention as it has been noted that inadequately controlled ear symptoms potentially lead to serious complications, including permanent hearing loss [11, 15, 16]. Therefore, in this study, we aimed to evaluate the characteristics, risk factor and clinical outcomes of MEE in EGPA patients.

## Methods

### Study participants and data collection

This is a case control study. Patients diagnosed and treated for EGPA from January 1995 to November 2018 were retrospectively identified from the prospectively collected EGPA registry data at Samsung Medical Center (a tertiary 1,997-bed referral hospital in Seoul, South Korea). EGPA was diagnosed according to ACR criteria [2]. Patients or who did not receive EGPA treatment or those with no follow-up data were excluded. All patients were treated under the same protocol to control the vasculitis activity of EGPA; 6 to 12 cycles of initial intravenous cyclophosphamide therapy when there was a major organ involvement (cardiovascular, gastrointestinal, renal, or central nervous system) or when peripheral motor neuropathy was confirmed by specialists via neurological examination and nerve conduction study using standard techniques [17]. After completion of intravenous cyclophosphamide therapy, a maintenance therapy with oral corticosteroids (prednisolone 5–15 mg per day) was administered. The oral corticosteroids were gradually tapered monthly when blood eosinophil level was ≤ 1500/μL and when there were no evident symptoms/signs of vasculitis activity. The oral corticosteroid dose was determined by individual clinical course of each patient. The Institutional Review Board (IRB) of Samsung Medical Center approved this study (IRB no. 2019-07-032) and waived informed consent due to the retrospective nature.

### Evaluation and management of the middle ear effusion

**Fig. 1:**
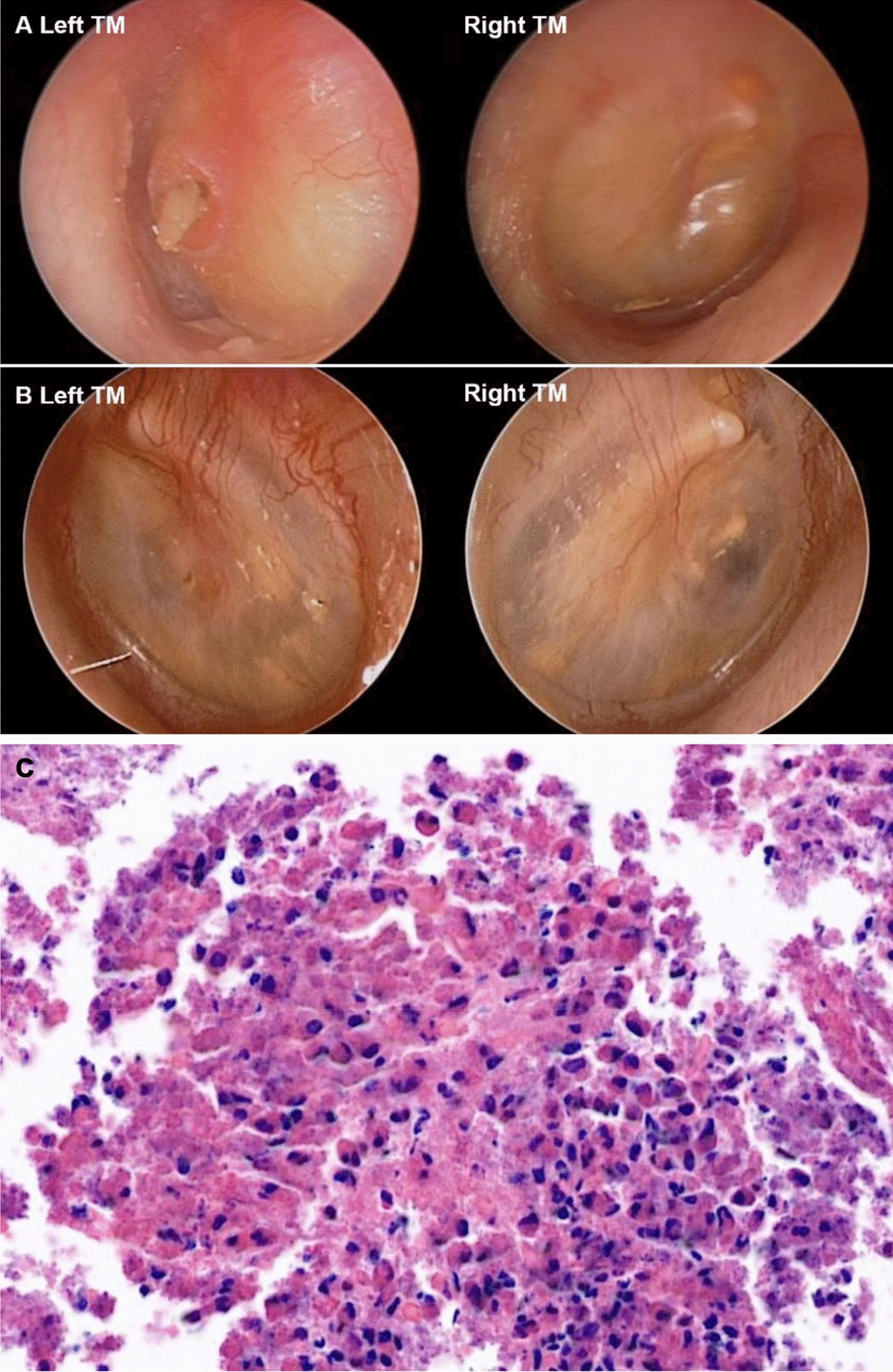
A representative case of MEE in EGPA patient; otomicroscopic image of MEE (**A**) before and (**B**) after increasing the maintenance dose of steroid, and (**C**) microscopic findings of eosinophil-rich middle ear discharge which were obtained through tympanic membrane perforation. EGPA, eosinophilic granulomatosis with polyangiitis; MEE, middle ear effusion

Patients with ear symptoms (ear fullness, ear discharge, tinnitus or hearing loss) at regular outpatient visits were referred to otologists and evaluated for MEE. When a thickened, bulging and pale tympanic membrane with a viscous or gelatinous secretion in the middle ear cavity was observed by otomicroscopy, samples from middle ear effusion or middle ear mucosa were collected through the tympanic membrane perforation or by myringotomy. The samples were fixed in formalin, embedded in paraffin, and then stained with hematoxylin and eosin to be observed under a light microscope for histological confirmation of the presence of the accumulation of eosinophils (Fig. 1) [18]. The cell count analysis for eosinophils were not possible in this study as the samples were often too thick and dense.

Patients with MEE underwent myringotomy/ventilation tube (VT) insertion and/or intratympanic steroid injection to control ear symptoms at the discretion of otologists. Although the choice of MEE treatment differed according to each patient’s situation, a myringotomy/VT insertion was performed to control MEE, and then intratympanic steroid injection was performed if there was no improvement. The dose of maintenance oral corticosteroids was increased upon agreement of both allergists and otologists.

### Outcome measurements

The main purpose of this study was to identify the factors associated with MEE in EGPA patients. Data on patient demographics and EGPA treatment-related features were complemented by a review of electronic medical records. Age, sex, fulfilled criteria of EGPA, involved major organs (heart, brain, lung, gastrointestinal tract or kidney) at diagnosis, and treatment details of EGPA were prospectively collected in the EGPA registry. In patients with MEE, the following data were obtained from electronic medical records to identify the factors associated with MEE: onset and type of ear symptom, previous history of MEE before a diagnosis of EGPA, severity and type of hearing loss (if present), and the treatment regimen for MEE. The last follow-up data were retrieved on March 12, 2021.

Patients with MEE were periodically evaluated by otomicroscopy for improvement or aggravation of MEE and audiometry for accompanying hearing loss, if present. Hearing levels were evaluated using pure-tone audiometry (PTA; Amplifon, Plymouth, MN, USA) in a sound-proof chamber. Hearing loss was defined based on pure-tone average, calculated using thresholds at 0.5, 1, 2, and 4 kHz, according to the National Institute on Deafness and Other Communication Disorders. Functionally normal hearing was defined as a PTA level < 15 dB.

### Statistical analysis

Data are reported as numbers with percentages in parentheses for categorical variables and as medians with interquartile ranges (IQRs) in parentheses for continuous variables. Categorical variables were compared using Pearson’s chi-square test or Fisher’s exact test, and the Mann–Whitney *U* test was used to compare continuous variables. The difference in eosinophil count before and onset of MEE was compared using the paired *t*-test. Hazard ratios (HRs) and 95% confidence intervals (CI) for the risk of MEE were calculated using the Cox proportional-hazard model. Multivariable analysis for MEE included clinically important variables or statistically significant variables in the univariate analysis: age, sex, previous history of MEE before diagnosis of EGPA, PNS abnormalities at initial diagnosis, blood eosinophil count at initial diagnosis, major organ (heart, brain, lung, gastrointestinal tract and kidney) involvement (any *vs* none), maintenance steroid cessation after vasculitis activity was controlled (tapered off *vs* not tapered), time of ear symptom after initial systemic therapy (< 6 months *vs* ≥ 6 months) [19]. All tests were two-sided, and a *P* -value < 0.05 was considered statistically significant. All analyses were performed using Stata (version 15.0; Stata Corporation, College Station, TX, USA).

## Results

### Patient characteristics

**Fig. 2:**
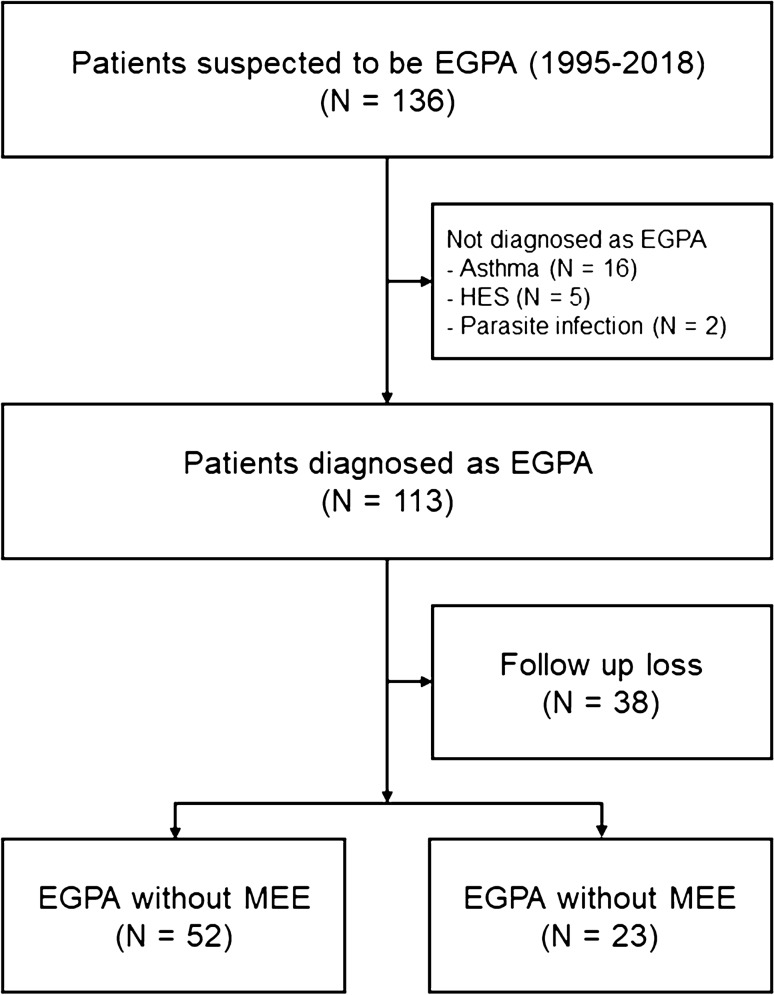
Flow chart of study patients. EGPA, eosinophilic granulomatosis with polyangiitis; HES, hypereosinophilic syndrome; MEE, middle ear effusion

From January 1995 to November 2018, 136 subjects were referred in suspicion of EGPA, and 113 patients were diagnosed as EGPA. After excluding patients with no treatment or follow-up data, 75 patients were included as study population (Fig. 2). The baseline characteristics of the study patients are summarized in Table 1. During the median follow-up of 9.9 years (IQR 6.3–13.1 years), 23 patients (30.7%) had MEE. The median age was 45 years (IQR, 35–55 years); 40 years (IQR, 33–51 years) for patients in the MEE group and 47 years (IQR, 35–59 years) for patients in the no MEE group (*P* = 0.085). Female patients comprised 52.0% of the study population.

**Table 1:** Baseline characteristics of study patients

Characteristics	Total (N = 75)	MEE ( +) (N = 23)	No MEE (N = 52)	*P*-value
Age, years	45 (35–55)	40 (33–51)	47 (35–59)	0.085
Sex				0.602
Male	36 (48.0)	10 (43.5)	26 (50.0)	
Female	39 (52.0)	13 (56.5)	26 (50.0)	
Concurrent at diagnosis				
Asthma	73 (97.3)	23 (100)	50 (96.2)	1.000
Peripheral neuropathy	56 (74.7)	16 (69.6)	40 (76.9)	0.499
PNS abnormalities	56 (74.7)	20 (87.0)	36 (69.2)	0.104
Extravascular eosinophils	50 (66.7)	15 (65.2)	35 (67.3)	0.859
Peripheral eosinophilia	38 (50.7)	13 (56.5)	25 (48.1)	0.500
Major organ involvement				
Lung	36 (48.0)	12 (52.2)	24 (46.2)	0.630
Heart	11 (14.7)	2 (8.7)	9 (17.3)	0.486
Gastrointestinal tract	9 (12.0)	3 (13.0)	6 (11.5)	1.000
Kidney	2 (2.7)	0	2 (3.6)	1.000
Brain	2 (2.7)	0	2 (3.6)	1.000
Laboratory findings^a^				
Eosinophil counts (/μL)	4430 (1746–7110)	4892 (1791–7198)	3875 (1381–7000)	0.861
≥ 5000 /μL	33 (44.0)	8 (34.8)	25 (48.1)	0.285
ESR (mm/h)	21 (10–39)	28 (18–57)	16 (8–39)	0.044
CRP (mg/dL)	0.6 (0.1–2.1)	1.2 (0.1–3.6)	0.5 (0.1–1.9)	0.048
ANCA	10 (13.9)	0	10 (18.5)	0.024
Total IgE (kU/L)	397 (130–1236)	710 (281–1596)	396 (92–943)	0.740
ECP (ug/L)	52 (17–201)	45 (17–114)	59 (17–201)	0.861
Systemic treatment regimen				0.778
Cyclophosphamide	57 (76.0)	17 (73.9)	40 (76.9)	
Steroid only	18 (24.0)	6 (26.1)	12 (23.1)	

Data are presented as number (percentage) or median (interquartile range)

ANCA, Antineutrophil cytoplasmic antibody; CRP, C-reactive protein; ECP, Eosinophil cationic protein; EGPA, Eosinophilic granulomatosis with polyangiitis; ESR, Erythrocyte sedimentation rate; MEE, Middle ear effusion; PNS, paranasal sinus

^a^Missing numbers are as follows; 2 patients had no data for ESR and ANCA, 4 for CRP, 16 for total IgE, and 21 for ECP

At the time of diagnosis, 73 (97.3%) patients had asthma. Peripheral neuropathy was presented in 56 (74.7%) patients, PNS abnormalities in 56 (74.7%), extravascular eosinophils on biopsy specimen in 50 (66.7%), peripheral eosinophilia (> 10%) in 38 (50.7%). The most common organ involvement was lung in 36 (48.0%) patients, followed by heart in 11 (14.7%), gastrointestinal tract in 9 (12.0%), kidney in 2 (2.7%), and brain in 2 (2.7%). The median blood eosinophil counts at the time of initial diagnosis of EGPA did not differ between the groups (MEE: 4892/μL; no MEE: 3875/μL; *P* = 0.861), and there were no association with MEE when the blood eosinophil count was categorized by ≥ 5000/μL (MEE: 34.8%; no MEE: 48.1%; *P* = 0.285), which is the currently widely accepted cut-off level for severe hypereosinophilia. Compared with the patients in the no MEE group, the patients in the MEE group had a significantly higher level of inflammatory markers, ESR (MEE: 28 mm/hr *vs*. no MEE: 16 mm/hr, *P* = 0.044), and CRP (MEE: 1.2 mg/dL *vs*. no MEE: 0.5 mg/dL, *P* = 0.048). Compared with the 10 (18.5%) patients with ANCA positivity in the no MEE group, none in the MEE group showed positive for ANCA (*P* = 0.024). To control the vasculitis activity, 57 patients (76.0%) were treated with intravenous cyclophosphamide therapy and 18 patients (24.0%) received only steroids. Overall, demographic data, clinical manifestations, or treatment regimens were not statistically significantly different between the MEE and no MEE groups.

### Otologic characteristics of MEE patients

**Fig. 3:**
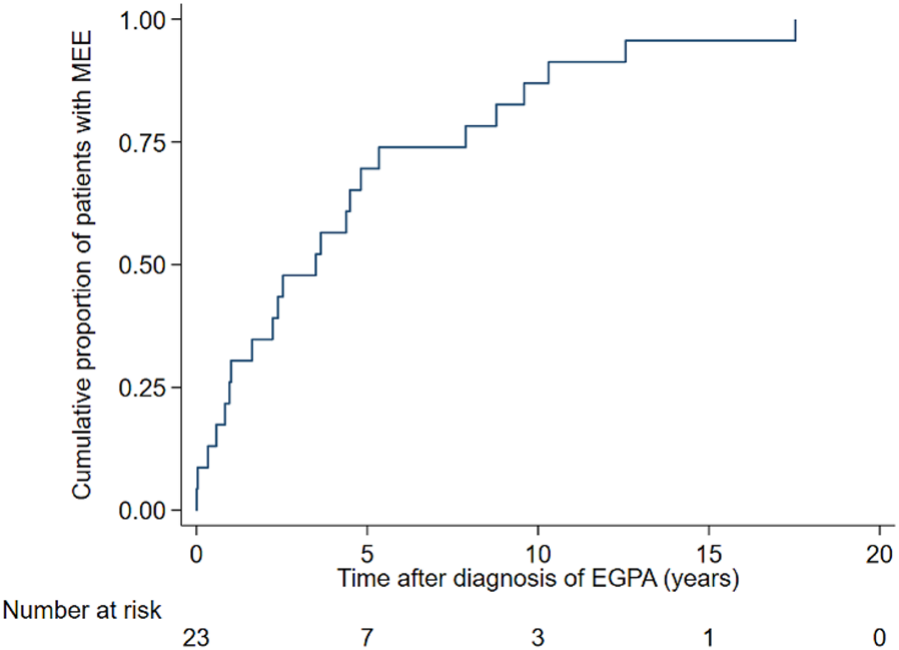
Kaplan–Meier curves for the occurrence of MEE after EGPA diagnosis. EGPA, eosinophilic granulomatosis with polyangiitis; MEE, middle ear effusion;

The otologic characteristics of patients in the MEE group are presented in Table 2. The median age at onset of ear symptoms was 45 years (IQR 35–65 years). Eighteen (78.3%) patients developed ear symptoms 4 years (IQR 1.8–7.2 years; Fig. 3) after the diagnosis of EGPA, while 5 (21.7%) had symptoms at the time of diagnosis. Four (17.4%) patients were receiving more than 10 mg of oral prednisolone as the maintenance steroid dose before the onset of MEE. Nine (39.1%) patients received > 5 mg but < 10 mg of oral prednisolone, and 3 (13.0%) received < 5 mg. Seven (30.4%) were steroid-free at the onset of ear symptoms.

**Table 2:** Otologic characteristics of patients with MEE (n = 23)

Characteristics	Median (IQR) or No. (%)
Age at onset of ear symptoms, years	45 (35–65)
Time from EGPA diagnosis to ear symptoms, years	3.6 (1.0–7.9)
History of MEE before diagnosis	5 (21.7)
Maintenance steroid dose before ear symptoms	
Prednisolone ≥ 10 mg	4 (17.4)
Prednisolone 5–10 mg	9 (39.1)
Prednisolone < 5 mg	3 (13.0)
None	7 (30.4)
Types of ear symptom^a^	
Ear fullness	16 (69.6)
Hearing loss	12 (52.2)
Tinnitus	7 (30.4)
Otorrhea	6 (26.1)
Otalgia	4 (17.4)
Severity of hearing loss (n = 15)	
Left	
Normal^b^	8 (53.3)
Mild hearing loss	5 (33.3)
Moderate hearing loss	2 (13.3)
Right	
Normal^b^	6 (40.0)
Mild hearing loss	5 (33.3)
Moderate hearing loss	4 (26.7)
Type of hearing loss (n = 12)	
Conductive hearing loss	10 (83.3)
Mixed hearing loss	2 (16.7)
Treatment regimen for MEE	
Myringotomy/VT insertion + IT injection + systemic steroid dose increase	7 (30.4%)
Myringotomy/VT insertion + systemic steroid dose increase	3 (13.0%)
IT injection + systemic steroid dose increase	1 (4.3%)
Steroid dose increase	6 (26.1%)
Myringotomy/VT insertion	3 (13.0%)
No treatment	3 (13.0%)
Recurrence of the ear symptoms	9 (39.1)

Data are presented as number (percentage) or median (interquartile range)

EGPA, Eosinophilic granulomatosis with polyangiitis; IT, Intratympanic; MEE, Middle ear effusion; VT, Ventilation tube

^a^Some patients complained of more than one symptom

^b^Functionally normal hearing classified based on PTA ≤ 15 dB

**Fig. 4:**
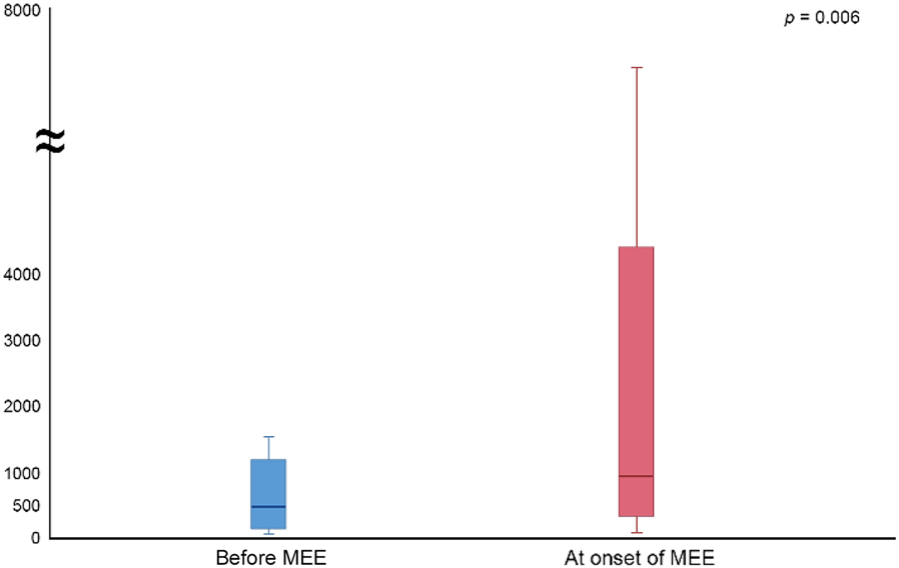
Change in blood eosinophil count before MEE and at onset of MEE. EGPA, eosinophilic granulomatosis with polyangiitis; MEE, middle ear effusion

The most common symptom was ear fullness in 16 (69.6%) patients, followed by impaired hearing in 12 (52.5%), tinnitus in 7 (30.4%), otorrhea in 6 (26.1%), and otalgia in 4 (17.4%). Among the 15 (65.2%) patients who underwent audiometry under suspicion of hearing loss, 12 (12/15, 80.0%) were confirmed, with both ears affected in 8 (8/12, 75.0%). Conductive hearing loss was observed in 10 (10/12, 83.3%) patients and mixed type of hearing loss in 2 (2/12, 16.7%) patients. The blood eosinophil count was significantly higher at the onset of MEE, compared to before MEE (before: 463/μL vs at onset: 919/μL, *P* = 0.006) (Fig. 4).

### Risk factors and clinical course of MEE

Factors associated with MEE in EGPA patients were evaluated (Table 3). In multivariable regression analysis, major organ involvement at diagnosis (adjusted hazard ratio [aHR] 65.4; 95% confidence interval [CI], 1.50—2838.39; *P* = 0.030], early onset of ear symptom after systemic therapy (< 6 months) (aHR 40.0; 95% CI, 1.35—1183.43; *P* = 0.033) and continuing the maintenance steroid without cessation (aHR 8.59; 95% CI, 1.13–65.42; *P* = 0.038) were independently associated with a risk of MEE, after adjusting for age, sex, previous history of MEE, PNS abnormalities at diagnosis, eosinophils count at diagnosis, ESR/CRP level at diagnosis, major organ involvement at diagnosis, cessation of maintenance steroids, and early onset of early symptom after systemic therapy.

**Table 3:** Risk factors associated with occurrence of MEE in EGPA patients

Variables	Crude HR (95% CI)	*P*-value	Adjusted HR (95% CI)	*P*-value
Age, years	0.97 [0.94–1.01]	0.157	1.05 [0.97– 1.13]	0.206
Females (vs males)	0.72 [0.30–1.70]	0.442	22.7 [0.87–593.88]	0.061
History of MEE before diagnosis	2.82 [1.01–7.88]	0.048	1.31 [0.13–13.38]	0.819
PNS abnormalities at diagnosis	2.48 [0.73–8.50]	0.147	2.8 [0.01–691.93]	0.712
Major organ involvement at diagnosis	1.89 [0.73–4.91]	0.190	65.44 [1.50–2838.39]	0.030
Eosinophil count at diagnosis	1.00 [0.99–1.00]	0.985	1.00 [0.99–1.00]	0.077
ESR level at diagnosis	1.02 [1.00–1.03]	0.099	1.02 [1.00– 1.05]	0.062
CRP lever at diagnosis	1.03 [0.96–1.12]	0.417	1.00 [0.74–1.36]	1.000
Continuing the maintenance steroid without cessation	0.48 [0.18–1.25]	0.132	8.59 [1.13– 65.42]	0.038
Early onset of ear symptom after systemic therapy (< 6 Months)	4.53 [1.42–14.47]	0.011	40.0 [1.35–1183.43]	0.033

CI, Confidence interval; EGPA, Eosinophilic granulomatosis with polyangiitis; HR, Hazard ratio; MEE, Middle ear effusion; PNS, Paranasal sinus

**Fig. 5:**
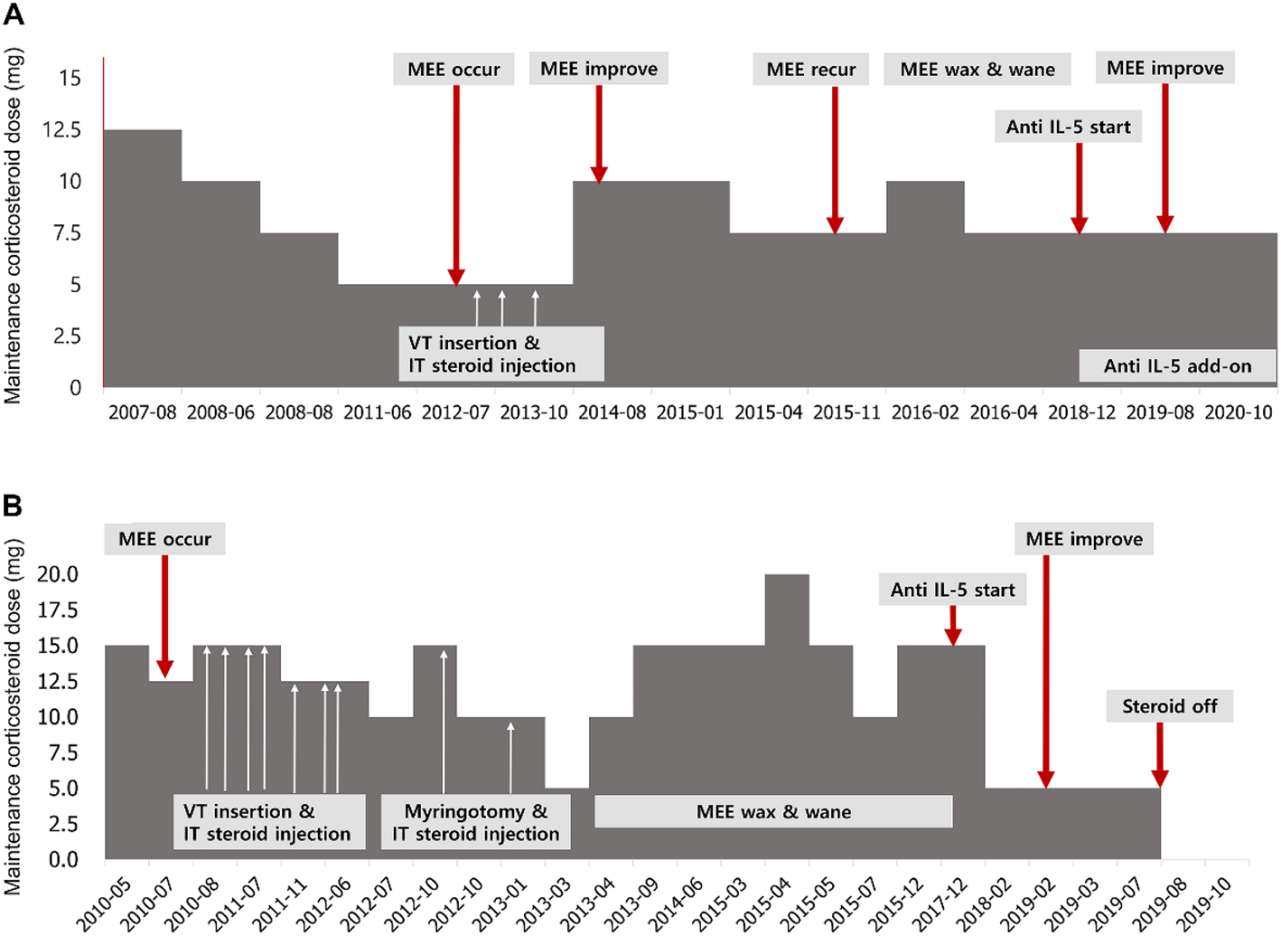
Serial changes in otologic symptoms and use of steroid/anti-IL-5 in 2 EGPA patients with recurrent MEE. **(A)** Patient A developed MEE while tapering maintenance steroids and **(B)** Patient B had MEE at the time of EGPA diagnosis. VT insertion/myringotomy and IT injections temporarily improved symptoms but steroid maintenance dosage had to be eventually increased. MEE waxed and waned based on steroid dose, which was better controlled after addition of anti-IL-5, without any events requiring additional steroids. *EGPA, eosinophilic granulomatosis with polyangiitis; MEE, middle ear effusion; VT, ventilation tube; IT, intratympanic*

To control the ear symptoms, myringotomy/VT insertion was performed in 10 (43.5%) patients (Table 2). Intratympanic steroid injection was administered in 8 (34.8%) patients; 7 (30.4%) had myringotomy/VT insertion with intratympanic injection and systemic steroid dose increase while one patient (4.3%) had intratympanic injection with systemic steroid dose increase. Six (26.1%) patients were treated by only increasing the systemic steroid dose and 3 (13.0%) were treated with external drainage only. Three (13.0%) patients had mild symptoms and were followed up without treatment. Nine (39.1%) patients with MEE had recurrent episodes (≥ 2) of MEE during follow-up. MEE mostly recurred within 5 years after diagnosis of EGPA (Fig. 3). Among the nine patients with recurrent MEE, two (22.2%) who could afford treatment received mepolizumab, which was the only anti–interleukin (IL)-5 agent available in Korea at the time (Fig. 5). Neither patient showed any other signs of active systemic vasculitis or other organ involvements after receiving systemic immunosuppressant therapy, but both had recurrent episodes of MEE whenever an attempt was made to taper the maintenance steroid dose. Both patients received VT insertion/myringotomy and intratympanic injections which temporarily improved ear symptoms, but eventually required increment of the maintenance steroid dose to control MEE, repeatedly. Mepolizumab, an anti–IL-5 agent, was used as a steroid salvage therapy. The indication of anti-IL-5 agent for these patients was solely due to MEE as these patients had no other symptoms/signs of active systemic vasculitis.

## Discussion

EGPA is a systematic disease that has a significant effect on ear, nose and throat. Compared with other involvements, otologic manifestations have been less understood [20]. In the present study, considerable number of patients had MEE that improved after a short-course of high-dose corticosteroids; however, approximately 40% experienced recurrent MEE afterwards and half of the patients had some degree of hearing loss. The use of an anti-IL5 agent in this study has showed its potential as steroid-salvaging therapy for MEE in EGPA.

Ashman et al*.* have reported that 39% of EGPA patients had ear symptoms at initial presentation [11]. This is in line with the findings from this study, which revealed that approximately 30% of EGPA patients experience at least one episode of MEE after systemic immunosuppressive therapy. Although a common feature, otologic manifestations are considered less serious than involvement of other major organs (e.g., heart, kidney), in which are directly associated with mortality [3]. As immunosuppressive therapy has improved the mortality of EGPA by suppressing vasculitis activity, it is now more important to manage chronic complications, such as MEE, to maintaining a good quality of life [21–24].

In MEE patients, otologic manifestations appeared mostly within median 4 years after diagnosis. Ear symptoms occur in 30% of patients after apparent suppression of vasculitis activity, even when maintenance steroids were completely tapered off. EGPA follows three distinct clinical phases: a prodromal phase characterized by asthma and rhinosinusitis, an eosinophilic phase marked by peripheral eosinophilia and organ involvement, and a vasculitic phase with clinical manifestations due to small-vessel vasculitis [2, 25]. The underlying pathophysiologic mechanism of MEE remains unclear, but otologic manifestations tend to occur more in the eosinophilic and vasculitic phases and presumably may occur out of sync with other systemic flares [9].

Major organ involvement at diagnosis of EGPA, early onset (< 6 months) of ear symptom after systemic therapy, and continuing the maintenance steroid were independently associated with MEE. Surprisingly, blood eosinophil counts at diagnosis were not associated with occurrence of MEE, although blood eosinophil counts at the onset of ear symptoms was higher than before MEE occurred, when the disease activity of EGPA was considered relatively stable. As the ear discharges were rich in eosinophils, peripheral eosinophilia may have been a trigger for tissue infiltration of eosinophils.

In several studies, neuritis of the vestibulocochlear nerve, or vasculitis of the internal auditory artery, was suggested to suddenly progress to hearing loss [12, 26]. However, the most frequent condition associated with MEE in the present study was conductive hearing loss, followed by mixed type hearing loss. Therefore, MEE can result in a mild-to-moderate conductive hearing loss [27].

For all EGPA patients, once the disease activity of vasculitis was controlled, the maintenance dose of oral corticosteroids was gradually tapered to as low a dose as possible or even tapered off entirely to minimize the long-term side effect of steroids. Nevertheless, patients with MEE had to temporarily increase the maintenance steroid dose. Local administration of steroids is reported to be effective for localized symptoms in MEE [28]. In the present study, however, intratympanic injection combined with external drainage via myringotomy/VT insertion showed temporal effectiveness, and the maintenance steroid eventually had to be increased in most patients (> 70%) to control MEE. In addition, Breslin et al. suggested that intratympanic injection of steroids in eosinophilic MEE may not be feasible, possibly due to the highly viscous nature of the effusion, which acts as a barrier to medication delivery [29].

Of these 23 MEE patients, 9 had recurrent episodes of MEE and had to increase and then again decrease steroids repeatedly to control the ear symptom. In these patients, a lifelong low-dose maintenance therapy of steroid may be necessary. The potential side effects of long-term steroid use cannot be ignored [30, 31] . Determining the optimal dose that does not induce recurrence of MEE and keeps the side effects of steroids to a minimum level remains a challenging problem. Recent advances in biological agents show they could be a novel therapeutic strategy in EGPA patients with MEE because eosinophils are thought to induce pathogenesis through tissue and vascular infiltration and inflammation through a variety of mediators [32–34]. Therefore, all patients with recurrent MEE were considered as a candidate for anti-IL-5 agents. However, biological agents for EGPA patients were not (and are still not) covered by health insurance in Korea and only two patients received anti–IL-5 agents as only they could afford the cost of the drugs. After administration of the anti–IL-5 agent, the number of events requiring a steroid increment due to MEE decreased, and one of the patients was able to taper off the steroids. The effectiveness of anti-IL-5 agents in EGPA should be carefully considered, as our experience is in only very small number. A well-designed prospective clinical trial is necessary to confirm the effect of anti–IL-5 agents.

The present study had a few limitations. First, racial differences must be considered. For instance, Asians show less positivity for ANCA. However, the main results agree with the previous publications from worldwide. Second, cell count analysis for eosinophils were not possible in this study as the samples were often too thick and dense, and the exact eosinophil counts could not be assessed. Last, the sample size may have not been large enough to detect other possible associations not reaching statistical significance in the multivariate analysis. However, EGPA itself is a rare disease, and to the best of our knowledge, this is the largest comparative study, with the longest follow-up, to focus on the otologic manifestations of EGPA. In addition, a close collaboration with an otologist was maintained and all cases were evaluated in an interdisciplinary manner.

## Conclusions

In conclusion, MEE is a common but frequently disregarded condition in EGPA which is often intractable. The maintenance dose of steroid for EGPA should be adequately adjusted to control the ear symptoms and to prevent progressive hearing loss. Novel biologic agents may have role in controlling MEE in EGPA.

## Data Availability

All data generated or analysed during this study are included in this published article.

## Abbreviations

ACR: American College of Rheumatology
ANCA: Antineutrophil cytoplasmic antibody
CI: Confidence interval
ECP: Eosinophil cationic protein
EGPA: Eosinophilic granulomatosis with polyangiitis
HR: Hazard ratio
IQR: Interquartile range
IT: Intratympanic
MEE: Middle ear effusion
PNS: Paranasal sinus
PTA: Pure-tone audiometry
VT: Ventilation tube
